# Supplementary material for: TRPML1-Induced Lysosomal Ca2+ Signals Activate AQP2 Translocation and Water Flux in Renal Collecting Duct Cells
Source: Int J Mol Sci. 2023 Jan 13;24(2):1647. doi: 10.3390/ijms24021647 (PMC9861594; doi:10.3390/ijms24021647)
Supplement: Supplementary file 1 [file ijms-24-01647-s001.zip › ijms-2112042-supplementary.pdf]

International Journal of *Molecular Sciences*

MDPI

Supplementary Materials

TRPML1-Induced Lysosomal Ca^{2+} Signals Activate AQP2 Translocation and Water Flux in Renal Collecting Duct Cells

Simona Ida Scorza [1,†], Serena Milano [1,†], Ilenia Saponara [1], Maira Certini [1], Roberta De Zio [1], Maria Grazia Mola [1], Giuseppe Procino [1], Monica Carmosino [2], Francesco Moccia [3], Maria Svelto [1] and Andrea Gerbino [1,*]

[1] Department of Biosciences, Biotechnologies and Environment, University of Bari Aldo Moro, 70125 Bari, Italy
[2] Department of Sciences, University of Basilicata, 85100 Potenza, Italy
[3] Department of Biology and Biotechnology Lazzaro Spallanzani, University of Pavia, 27100 Pavia, Italy
* Correspondence: andrea.gerbino@uniba.it; Tel.: +39-0805443334
† These authors contributed equally to this work.

Citation: Scorza, S. I.; Milano, S.; Saponara, I.; Certini, M.; De Zio, R.; Mola, M.G.; Procino, G.; Carmosino, M.; Moccia, F.; Svelto, M.; et al. TRPML1-Induced Lysosomal Ca^{2+} Signals Activate AQP2 Translocation and Water Flux in Renal Collecting Duct Cells. *Int. J. Mol. Sci.* **2023**, *24*, 1647. https://doi.org/10.3390/ijms24021647

Academic Editor: Frank Thévenod

Received: 5 December 2022
Revised: 4 January 2023
Accepted: 11 January 2023
Published: 13 January 2023

Copyright: © 2023 by the authors. Licensee MDPI, Basel, Switzerland. This article is an open access article distributed under the terms and conditions of the Creative Commons Attribution (CC BY) license (https://creativecommons.org/licenses/by/4.0/).

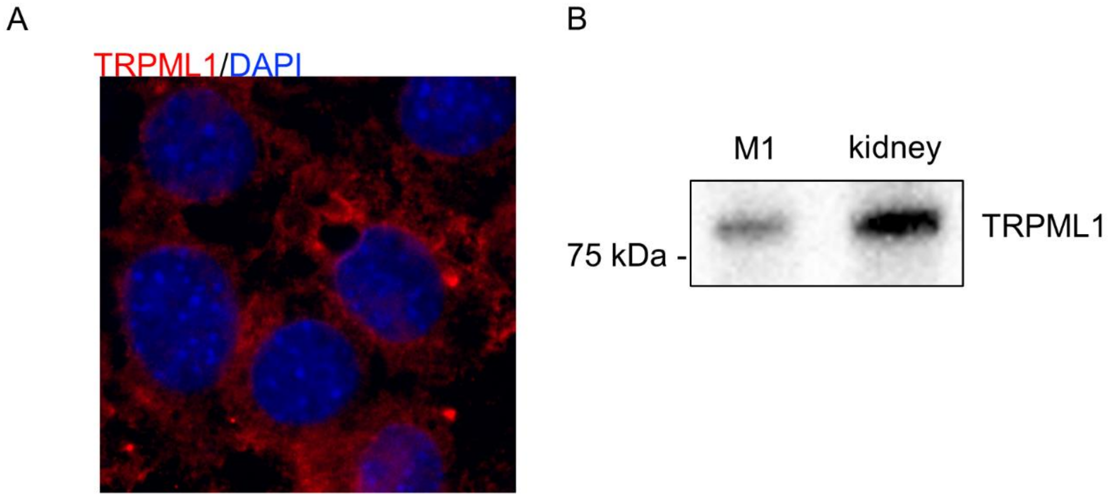

Figure S1. M1 cells express the lysosomal cation channel TRPML1. A) Representative immunofluorescence acquisition of the cation channel TRPML1 (red) merged with the nuclear dye DAPI (blue). B) Western blotting analysis of TRPML1 on M1 cells lysates (left) and total kidney homogenates (right).

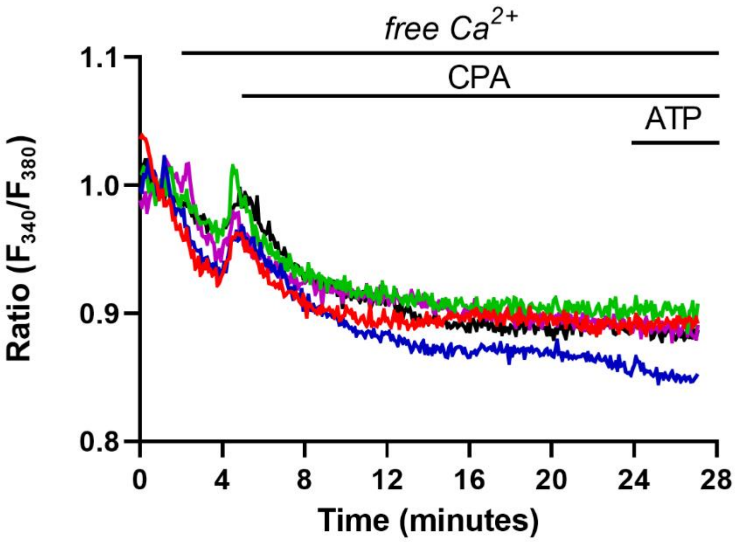

Figure S2. Pre-treatment with CPA empties the Ca2+ content of the ER. Ca2+ response of M1 cells loaded with Fura-2 to the addition of the Ca2+ mobilizing agent 100 µM ATP after 20 min pre-treatment with 40 µM CPA in free Ca2+ condition (*n* = 109/118 responsive cells, *m* = 3 experiments). Graph shows 5 representative traces.
